# The Confrontation between Ethnopharmacology and Pharmacological Tests of Medicinal Plants Associated with Mental and Neurological Disorders

**DOI:** 10.1155/2018/7686913

**Published:** 2018-07-02

**Authors:** Giovanna Felipe Cavalcante e Costa, Hisao Nishijo, Leonardo Ferreira Caixeta, Tales Alexandre Aversi-Ferreira

**Affiliations:** ^1^Federal University of Tocantins, Legal Amazonia, Brazil; ^2^System Emotional Science, Graduate School of Medicine and Pharmaceutical Sciences, University of Toyama, Toyama, Japan; ^3^Unit of Neuropsychiatry, Neuropsychology and Behavior Neurology (UNCO), Federal University of Goiás, Goiania, Brazil; ^4^Laboratory of Biomathematics, Department of Anatomy, Federal University of Alfenas, Alfenas, MG, Brazil

## Abstract

For neurological disorders, pharmacological tests have shown promising results in the reduction of side effects when using plants with known therapeutic effects in the treatment of some types of dementia. Therefore, the goals of this study are to gather data about the major medicinal plants used in the nervous system as described in ethnopharmacological surveys from South America and Brazil and to compare this data with the results from pharmacological tests on the active principles of those same plants found in the scientific literature. After collecting the data about each plant, their respective popular indication was compared with the results found through pharmacological tests. The discrepancy rate between the effects observed by ethnopharmacological and pharmacological methods in this study is greater than 50%. In conclusion, despite the importance of ethnopharmacological data, it is important to make comparisons with pharmacological tests for the same plants, since the pharmacological studies, although few, have shown a high rate of discrepancy in the results.

## 1. Introduction

The knowledge of medicinal plants for therapeutic purposes originated from indigenous tribal cultures [1–4] or ancient civilizations such as those once found in Iran, India, or China [1–3, 5–7] and was passed from generation to generation mostly by means of oral tradition. Presently, knowledge is commonly limited to a village and rural areas or by families isolated from urban centers [8]. Most likely, original information of plants used for therapeutic purpose underwent modifications through time. This was due to their discovery by trial and error over many generations and the oral transmission of information rather than through writing.

A previous study associated culturally propagated therapeutic effects of different medicinal plants obtained by ethnopharmacological/ethnobotanical means with those found in laboratory tests, showing approximately 66% discrepancy in the results [9]. Trading and distribution mistakes [10], similarity of plant names for different species [11], presence of impurities during preparation from other plants, insects, and mushrooms [12], and unexpected reactions and interactions with the active compounds [13] are all examples of commonly encountered problems in the therapeutic use of medicinal plants.

It is not suggested that the medicinal use of plants should be banned, decreased, or hampered. However, there is a need for each procedure to be evaluated by government agencies, institutions, and specialists who understand the therapeutic use of biodiversity in societies with an increasing interest in alternative treatments [6, 14, 15] or in populations with limited or no access to other types of therapeutic resources. Medicinal plant-based therapy may offer benefits, like decreased side effects [16–18], higher autonomy for individuals in caring for their own health [3], reduced or nonexistent costs, and easy access for social groups located in inaccessible areas or away from urban centers and for people in poor urban areas with limited or no access to a healthcare system [6, 14, 15, 19, 20]. Indeed, those groups rely on alternative therapeutic methods for their health care, especially those derived from local medicinal plants, which is a major issue in countries with higher income gaps.

Many ethnopharmacological surveys were performed in countries and regions representing the greatest biodiversity to identify plants used, with the aim of preserving the cultural heritage of the plant therapy [1–3, 5–7, 21, 22] and acquiring new active compounds for the pharmaceutical industry [8]. Brazil presents the largest biodiversity on the planet [23] and has a large amount of unexplored resources available for ethnopharmacological and herbal studies given that only 16% of Brazil's medicinal plants or just 8% of Brazilian national flora [24] has been evaluated for therapeutic potential [25]. This country represents around 47% of all territories of the South American continent.

Countries in South America present important data about medicinal plants, because of their specific locations in the Andean region, close/into the Amazon Forest [8] or the pampas. Indeed, the use of some medicinal plants was first found in the population in the Andes Ridge, in the pampas, Patagonia [10], or Brazilian's savanna (cerrado) [9]. Probably because of the large population or size, most of the studies in South America are found in Brazil, while ethnopharmacological studies are incipient in other countries in this continent [8, 10].

However, quality or reliability of medicinal plant effects cannot be ensured if ethnobotanical studies do not provide laboratory verification of the effects when prescribing compounds derived from those medicinal plants. Healthcare professionals and patients should note that studies about the correspondence or discrepancy between ethnopharmacological knowledge and laboratory tests for the same plant are lacking [9] and must be done for each class of drug.

There is a growing evidence from in vitro, animal, and clinical studies reporting that medicinal plants might be beneficial for treating various mental and neurological disorders including Alzheimer disease, depression, anxiety, and insomnia [363–366]. For neurological disorders, in particular, pharmacological tests have shown promising results in the reduction of side effects when using plants with known therapeutic effects in the treatment of some types of dementia [18, 22, 367–372]. Medicinal plants have been sought as an alternative therapy [18, 373–375] owing to the inefficacy of some industrial medications on certain diseases, such as degenerative ones. Examples are the use of* Melissa officinalis, Salvia officinalis, Ginkgo biloba, *and* Huperzia serrata *for treating the symptoms of Alzheimer disease [18, 373–375].

The problem is that, especially in developing and/or populated countries, people rely on medicinal plants as primary healthcare [376]. The situation is true for mental and neurological disorders. Patient complaints associated directly or indirectly with neurological or neuropsychiatric disorders, such as headache, insomnia, amnesia, anxiety, or depression, are very common [146, 298, 377, 378], and the use of medicinal plants for these purposes is very frequent in populated countries such as Brazil, India, and China [1–3, 5–7, 22] but without support of adequate pharmacological tests.

Considering the errors in the use and sale of alternative medicines as a whole, we hypothesize that the same errors could happen with plants that act directly on the nervous system. Therefore, the goal of this study is to gather data about the major medicinal plants used in the neural system, as described in ethnopharmacological surveys from South America like in Brazil and compare this data with the results from pharmacological tests on the active principles of those same plants found in the scientific literature. Specifically, this study intends to present reliable data for the use of medicinal plants in primary healthcare and assisting conventional treatments of neurological disorders.

## 2. Materials and Methods

This study was done through literature review of ethnopharmacological surveys on the medicinal plants used by groups in South America (with emphasis on Brazil) found in academic databases (MEDLINE, LILACS, Scopus, SciELO, Google Academic, and Elsevier). The terms searched were ethnobotanical studies, medicinal plants, ethnopharmacology, neural system, South America, and Brazil. The search was restricted to the most recent and classical articles/books written in Portuguese, English, or Spanish. After collecting the data about each plant, their respective popular indication was compared with the results found through pharmacological tests.

For the first phase, 55 ethnobotanical survey articles were selected and then the most commonly used plants by the population for treating neural system disorders were identified. A table was prepared with data regarding family, scientific name, part of the plant utilized, preparation method, indications, and comparison with pharmacological tests.

In the second phase, 181 articles in which pharmacological tests had been performed with the chosen plants were selected. Unfortunately, scientific tests for the proposed indication or toxicity for all the plants could not be found.

Statistical analysis was done using central tendency measures such as modal frequency.

## 3. Results

**Figure 1:**
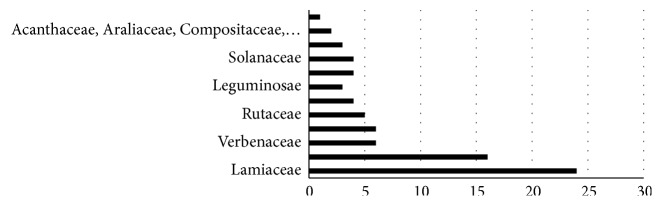
The cited families of medicinal plants according to popular knowledge.

Data on South American medicinal plants that act on the nervous system was summarized by family, scientific name, part of the plant utilized, preparation method, indications, and comparison with pharmacological tests (Table 1). The most cited families were Lamiaceae (24/138), Asteraceae (16/138), and Verbenaceae (6/138), representing 33.7% of the medicinal plants analyzed (Figure 1).

**Figure 2:**
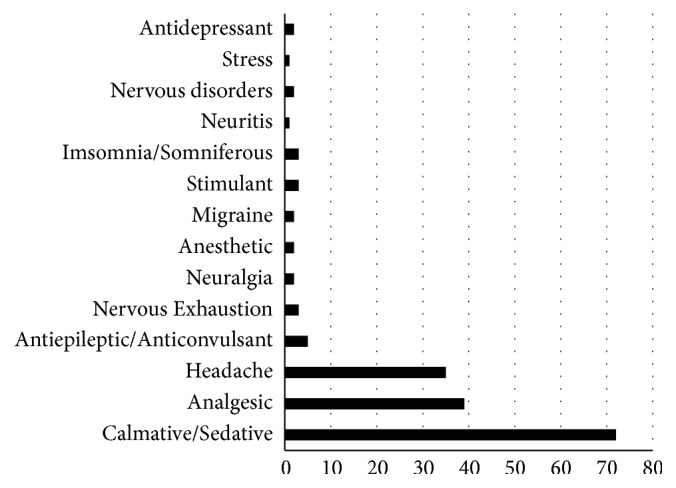
The indications for use of the medicinal plants according to popular knowledge.

The most common indications, according to ethnopharmacological surveys, were calmative/sedative (72/167), analgesic (39/167), and headache (35/167), representing 86,2% of all indications (Figure 2).

**Figure 3:**
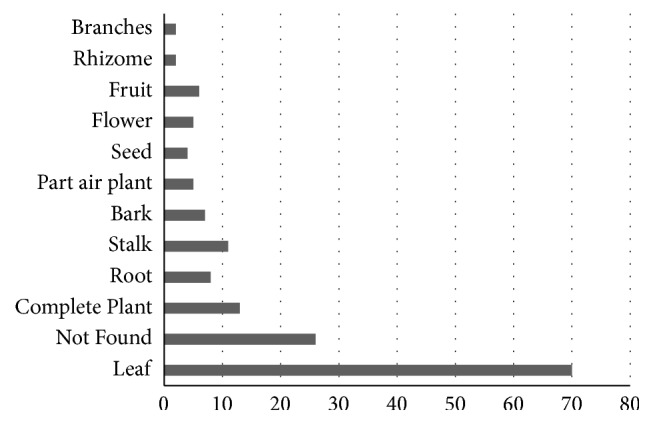
Part of plants used for indications according to the popular knowledge.

Ethnobotanical surveys revealed that the leaves (70/160) and the whole plant (13/160) amounted to 51.7% of all plant parts most commonly used, but, in 18% of the studied plants, there were no citations about the used part for making medicines (Figure 3).

**Figure 4:**
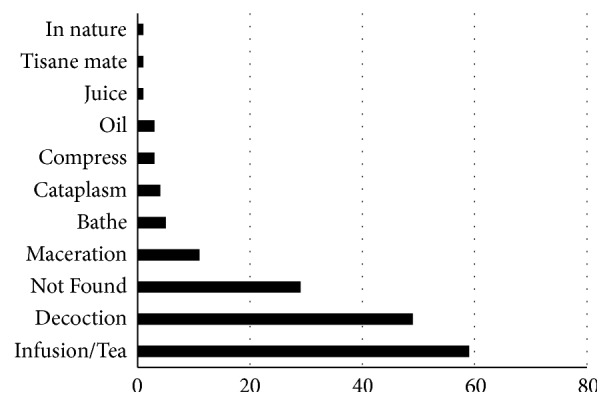
Preparation methods cited by population for medicinal plants.

The most common preparation methods provided in the surveys were infusion (59/167) and decoction (49/167), representing 63.7% of all the methods (Figure 4).

**Figure 5:**
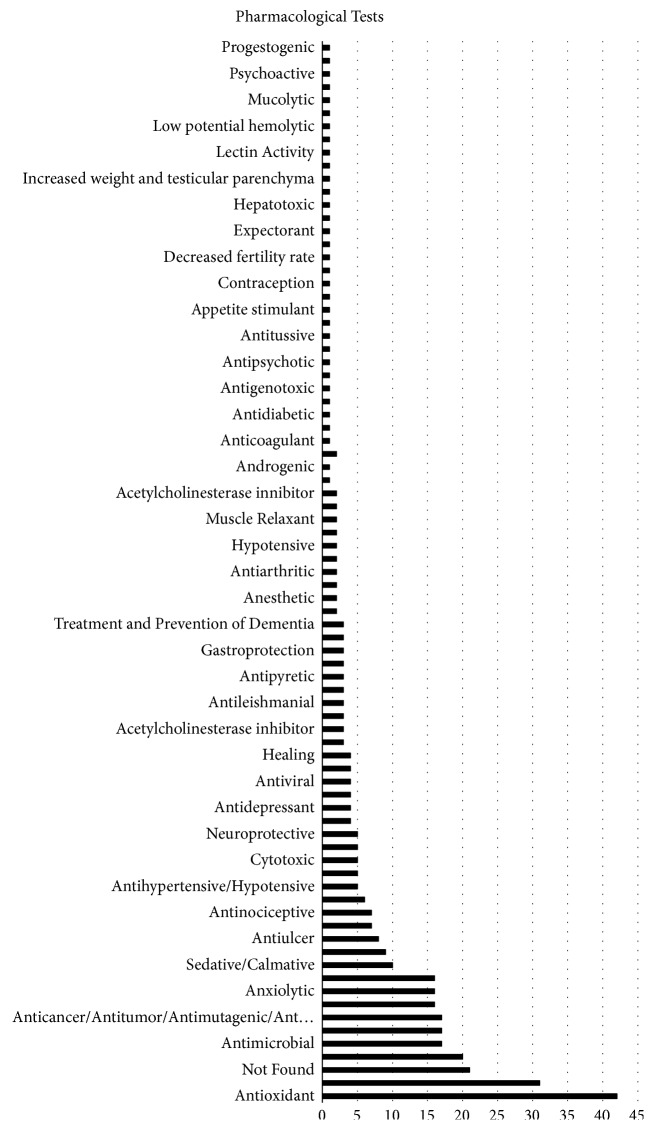
Attributed effects of the medicinal plants according to popular knowledge.

Common effects attributed to the plants in the ethnopharmacological surveys were antioxidant (42/401), anti-inflammatory (31/401), antibacterial (20/401), and antimicrobial (17/401), totaling 31.9% (Figure 5).

**Figure 6:**
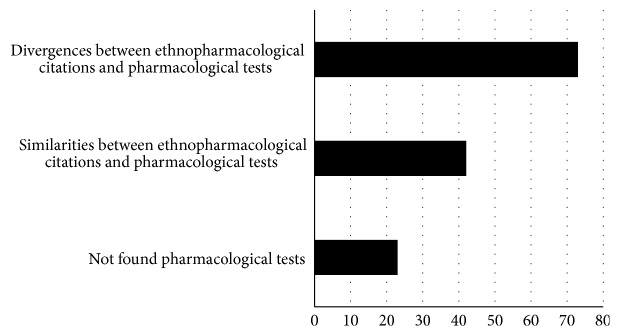
Comparison between ethnopharmacological data and pharmaceutical tests for the same plants and compounds.

Comparison between ethnopharmacological data and pharmaceutical tests for the same plants and compounds found differences in 52.9% (73/138) of the cases and similarities in 30.4% (42/138) (Figure 6). No pharmacological tests were found for 16.9% (23/138) of the plants mentioned in the ethnopharmacological surveys (Table 1).

Table 1 shows a list of the medicinal plants analyzed in this study. The pharmacological effects including “anticonvulsant” and “anxiolytic” were considered to correspond to “calmative” in medicinal effects cited by population since both effects are attributed to the same action in the neural system, that is, inhibitory action. Furthermore, the pharmacological effect “anti-inflammatory” was also considered to correspond to “analgesic” in medicinal effects cited by population since anti-inflammatory agents are effective in treating pain diseases.

## 4. Discussion

The most frequent indications of medicinal plant use for neural system disorders in our survey (i.e., calmative, analgesic, headache, and insomnia) are associated with the most common occurrences seen in medical practice [7, 36, 47, 55, 68, 77, 104, 132, 235, 258] (Figure 2).

The plant families analyzed (Lamiaceae and Asteraceae) are in accordance with general ethnobotanical studies [4, 7, 379–382] (Figure 1), as well as the most utilized plant parts (leaves) [1, 7, 379, 383, 384], and preparation methods (infusion and decoction) [7, 253, 379, 383, 384] (Figure 4).

Despite that, the frequency of effects observed by most pharmacological tests does not coincide with those reported for the same plants when analyzed by ethnopharmacological means, (i.e., antioxidant, anti-inflammatory, antibacterial, and antimicrobial), demonstrating a high discrepancy between proven and popularly mentioned effects (Figure 6).

It is important to remember that results of pharmacological tests were not found for all the plants mentioned in the ethnopharmacological studies, although those represent a small minority (16.9%) (Figure 5).

The discrepancy rate between the effects observed by ethnopharmacological and pharmacological methods in this study is in agreement with a previous study [9] and, in both cases, a disagreement of over 50% was found. This data indicates the need for better control in the use of medicinal plants as a whole, especially in countries with a large proportion of economically backward population where such therapy is most common, such as China, India, and Brazil. However, there are possibilities that scientific studies are not enough or they are missing to corroborate the ethnopharmacological activities.

Tables like the one produced in this study can be used as a basis for the indication of medications for health professionals working in the neural area who choose to substitute alternative therapies with conventional methods. The tables can be used to maintain the patient's health and help make these treatments more accessible to people of all economic levels [385], bring medical practice closer to the care of cultural groups [386], and expand the idea of wholeness in healthcare.

Performing pharmacological tests in the medicinal plants mentioned in ethnopharmacological studies will help avoid prescription errors based only on popular knowledge, which, despite the importance, exhibits extensive methodological shortcomings from its propagation through generations (see Introduction). Although the pharmacological tests cannot solve problems related to contamination during preparation and/or mistakes when identifying plants by unskilled people, performing those tests would decrease the problems caused by adverse effects and wrong prescriptions.

Neurological disorders present complex etiologies often with aggravating social influences, requiring special care when making prescriptions; many critically ill patients are secluded from society and require medical monitoring and medications derived from modern pharmaceutical technology since indications for complex etiologies like dementias were not addressed in the ethnopharmacological articles analyzed in this study.

In conclusion, despite the importance of ethnopharmacological data, it is important to make comparisons with pharmacological tests for the same plants, since the pharmacological studies, although few, have shown a high rate of discrepancy in the results, nevertheless, to be important to cite that the scientific studies could not be enough, or are missing, to corroborate the ethnopharmacological activities. Tables containing the plants names and their effects according to pharmacological tests should be consulted by health professionals before prescribing those medications. No medicinal plants were mentioned in ethnopharmacological data for treating complex etiology neural disorders such as dementia, indicating the need for new studies of broader geographical amplitude and pharmaceutical classes all around the world. Emphasis of these studies should occur in developing countries in order to decrease prescription errors associated with medicinal plants and increase the coverage of plant-based therapy for the global population while prioritizing people in need.

## Figures and Tables

**Table 1:** Family names, forms of preparation, used part of the plants, medicinal effects cited by population, the pharmacological effects tested for cited plants with the references, and the divergence between the cited effects by population and pharmacological tests.

Family Scientific name/common name	Forms of preparation/used part	Medicinal effects cited by population	Pharmacological tests	Divergences
Acanthaceae				

*Hygrophila tyttha *Leonard*/*Tame-male	Infusion/Part air plant	Calmative [26]	Anxiolytic effect, anticonvulsant and sedative [26]	No

*Justicia pectoralis *Jacq./Anador	Decoction/Leaf	Headache [27]	Anxiolytic and depressor Neural Central System [28], analgesic and anti-inflammatory [29], estrogenic, progestagenic and anti-inflammatory effects [30], antioxidant [31]	No

Alismataceae				

*Echinodorus grandiflorus *(Cham. & Schltdl.) Mich./Hat leatherback	Decoction/Leaf	Analgesic [21]	Anti-inflammatory and analgesic [32, 33], diuretic [33], antihypertensive [34, 35]	No

Amaranthaceae				

*Alternanthera paronychioides *St-Hil./Anador	Not found/Leaf, stalk	Analgesic [36]	Antioxidant [37]	Yes

Apiaceae				

*Apium graveolens *L.*/*Celery	Not found/Complete plant	Calmative [36]	Vasorelaxant and antihypertensive [38]	Yes

*Coriandrum sativum *L./Coriander	Infusion/Seed	Headache [39]	Antioxidant [40], anti-inflammatory [41], antibacterial [42], anxiolytic, sedative and muscle relaxant [43], antifungal [44], hypoglycemic, hypolipidemic and hepatoprotective [45], analgesic [46]	No

*Pimpinella anisum* L./Fennel	Infusion/Seed	Calmative [7, 27, 39, 47–49]	Antibacterial [50], neuroprotective and anticonvulsant [51], antiviral and immunostimulating [52], antioxidant [53], anticancer [54]	No

*Foeniculum vulgare*Mill.*/*Fennel	Decoction/Stalk	Headache and calmative [13, 36, 55–60]	Antimicrobial [61], diuretic [62], antihelminthic [63], antioxidant [64], anxiolytic [65]	No

Aquifoliaceae				

*Ilex paraguariensis*/Erva Mate	Infusion/leaves, branches	Stimulant [66]	Stimulant [66]	No

Araliaceae				

*Didymopanax macrocarpum *(C. & S.) Seem./ Five leaves	Compress, bathe/Leaf	Analgesic [67]	Not found	Not found

*Hedera helix*/Hiedra	Cataplasm/Leaf	Analgesic, neuritis, neuralgia [68]	Expectorant and antitussive [69], mucolytic and bronchodilator [70], anti-inflammatory [71]	No

Aristolochiaceae				

*Aristolochia esperanzae *O. Kuntze./Papo de peru, cipo-millhomem	Decoction/Complete plant	Analgesic [67]	Antiophidic activity [72], antimicrobial [73]	Yes

*Aristolochia gilbertii *Hook/Milhomem	Infusion/Root	Headache [7]	Not found	Not found

*Aristolochia melastoma *Manso ex. Duchtra/Capitãozinho	Decoction/Root, leaf	Sedative [67]	Not found	Not found

Asteraceae				

*Achillea millefolium *L./Ponta-alívio	Decoction/Complete plant	Calmative, analgesic [21, 36, 47, 57, 74, 75]	Immunostimulating [76]	Yes

*Achyrocline satureioides *D.C./Macela	Infusion/Flower	Sedative, calmative, headache [56, 67, 75, 77, 78]	Anticancer [79], calmative effect, anti-inflammatory and antispasmodic [80], antiviral [81]	No

*Artemisia absinthium *L./Losna	Decoction/Leaf	Analgesic [21, 82]	Anticancer [83], antifungal [84], antibacterial [85], antileishmanial [86]	Yes

*Artemisia camphorata *Vill./Camphor	Infusion/Leaves	Calmative [58] antiepileptic [87]	Not found	Not found

*Artemisia vulgaris *L./Artemisia	Not found	Headache [88]	Antifungal [89]	Yes

*Chamomilla recutita *(L.) Rauschter/Camomile	Infusion/Flower	Calmative, sedative [36, 39, 48, 57, 90–92]	Antibacterial and anti-inflammatory [92, 93], gastroprotection [94], antihyperglycemic and antioxidant [95]	Yes

*Chrysanthemum parthenium *Bernhadi/Artemisia	Decoction, infusion/Leaves	Calmative [78]	Not found	Not found

*Cynara scolymus *L./Artichoke	Not found	Calmative [74]	Diuretic [96], prolonged satiety sensation and hypoglycemic [97], antioxidant [98]	Yes

*Lactuca sativa *L./Lettuce	In nature, infusion/Leaves, root	Calmative, sedative [74, 99]	Antioxidant [100]	Yes

*Matricaria chamomilla L*./Camomile	Infusion/Leaves	Calmative [56, 75, 78, 101, 102]	Antibacterial and antioxidant [103]	Yes

*Mikania hirsutissima DC./*Cipó-cabeludo	Not found	Calmative [67, 104]	Antiophidic activity and antidiarrheal [105]	Yes

*Solidago chilensis Meyen*/Arnica	Compress/Complete plant	Analgesic [106]	Anti-inflammatory [107]	Yes

*Spilanthes oleracea/*Anestesiol	Not found	Anesthetic [108]	Peptic antiulcer and contraception [109]	Yes

*Tanacetum *sp./Macelinha	Decoction/Complete plant	Analgesic [23]	Not found	Not found

*Tanacetum vulgare L.*/Catinga-de-mulata	Decoction, maceration/Leaves	Analgesic [23]	Antibacterial and antifungal [110], antiviral [111], cytotoxic [112], treatment of infections caused by *Trypanosoma cruzi *and *Leishmania amazonensis *[113], immunomodulatory [114], antihelminthic [115]	Yes

*Vernonia *cf. *condensata *Baker./Boldo do chile	Infusion/Bark	Calmative [57]	Antitumor and anti-inflammatory [116], antioxidant [117]	Yes

Bignoniaceae				

*Anemopaegma arvense/*Catuaba	Infusion, decoction/Root, bark, leaves	Nervous exhaustion [118]	Increased weight and testicular parenchyma [119], antifungal [120]	Yes

Bombacaceae				

*Eriotheca candolleana *(K. Schum.)/Catuaba	Infusion/Root	Nervous exhaustion [121]	Not found	Not found

Boraginaceae				

*Cordia verbenacea *DC.*/*Maria preta	Bathe/Leaves	Analgesic [49]	Antimicrobial [122], anti-inflammatory [123]	No

Brassicaceae				

*Coronopus didymus *(L.) Smith/Mastruz	Maceration/Leaves	Analgesic [49]	Healing [124], anti-inflammatory [125]	No

Bromeliaceae				

*Tillandsia usneoides* (L.) L/Barba de velho	Not found	Antiepileptic [49]	Abortion [126], antiviral [127]	Yes

Buddlejaceae				

*Buddleja brasiliensis *Jacq./Verbasco	Infusion, cataplasm/Part air plant	Calmative [67]	Low potential hemolytic [128]	Yes

Burseraceae				

*Commiphora myrrha *(T. Nees) Engl/Myrrh	Infusion/Leaves	Calmative [49]	Antioxidant [117], analgesic [129]	Yes

Caesalpiniaceae				

*Bauhinia forficata *Link./Pata de vaca	Decoction/Leaves	Analgesic [23]	Antioxidant and increased liver glycogen [130], antimutagenic [131]	Yes

*Bauhinia rutilans Spruce ex. Benth*/Escada-de-macaco	Infusion/Part air plant	Analgesic [99]	Not found	Not found

Canellaceae				

*Capsicodendron dinissi *Occhioni/Pepper	Not found	Migraine [104]	Not found	Not found

Capparaceae				

*Cleome spinosa Jacq./*Mussambê	Infusion/Complete plant	Headache [132]	Cytotoxic [49], antioxidant [133], anti-inflammatory and antinociceptive [134]	No

Caprifoliaceae				

*Sambucus nigra *L./Elderberry	Decoction/Leaves	Analgesic [23, 49]	Anti-inflammatory and antioxidant [134], parasiticidal [135]	No

Chenopodiaceae				

*Chenopodium ambrosioides *L./Yerba Santa Maria	Maceration, infusion/Leaves, bark, seed	Analgesic, calmative [23, 48, 57]	Antitumor [79], hypotensive [136], antipyretic and anxiolytic [137]	Yes

Compositaceae				

*Baccharis trimera* (Less) D.C./Gorse	Infusion/Leaves	Headache [138]	Antiulcer and antioxidant [139], anti-inflammatory [140], anti-inflammatory and analgesic [141]	No

*Vernonia condensata *B./Boldo	Infusion, decoction/Leaves	Calmative [138]	Antioxidant [117], analgesic [142]	Yes

Cucurbitaceae				

*Cayaponia tayuya *(Vell.) Cogn./Taiuia	Infusion, decoction/Root	Neuralgia [67]	Hepatotoxic [142], anti-inflammatory [143]	Yes

Dilleniaceae				

*Davilla rugosa *Poir./Vine cabloco	Bathe/Root	Sedative [67]	Antioxidant [144], antiulcer [145]	Yes

Euphorbiaceae				

*Jatropha curcas *L.*/*Pião-bravo	Infusion/Seed	Headache [102, 132]	Acetylcholinesterase inhibitor [146], antibacterial, antioxidant and antitumor [147, 148], cytotoxic [149]	Yes

*Ricinus communis *L./Castor beans	Infusion/Leaves	Headache [77, 87]	Antimicrobial and anticancer [150], antimicrobial [151]	Yes

Fabaceae				

*Caesalpinia ferrea *Mart. ex. Tul./Pau ferro	Not found	Analgesic [57]	Nutritional supplementation of iron, zinc and manganese [152] anti-inflammatory and healing [153], antihyperglycemic [154], antimicrobial [155]	No

*Cajanus flavus De Candolle*/Andu beans	Infusion/Leaves	Headache [99]	Not found	Not found

*Erythrina falcata Benth*/Surina, mulungu	Not found	Sedative and antiepileptic [67, 104]	Depressant CNS [156]	No

*Indigofera anil/*Anil	Not found	Sedative [107]	Not found	Not found

*Indigofera suffruticosa *Mill./Anileira	Decoction, infusion/Complete plant	Sedative [67]	Anti-inflammatory [157], lectin activity [158], antiepileptic [159], antiparasitic [160]	Yes

*Pterodon emarginatus*/Sucupira	Infusion/leaves, fruit	Headache [120]	Antimicrobial [161–163], analgesic and anti-inflammatory [164]; antileishmanial, anticancer, hypoglycemic [165]	No

Ginkgoaceae				

*Ginkgobiloba*/Ginco	Decoction, infusion/Leaves	Vasodilator, brain dysfunction, dizziness and concentration and memory [160]	Treatment of Alzheimer disease [166], prevention of dementia [167], antioxidant, vasodilator, stimulant of SNC [168]	No

Geraniaceae				

*Pelargonium graveolens *L'Her/ Mauve smelling	Not found	Sedative [87]	Anxiolytic and antidepressant [159], antibacterial [169], hypoglycemic and antioxidant [170]	No

*Mimosa pudica *L./Dormideira	Infusion/Complete plant	Sedative [99]	Reduction of fertility [171], hepatotoxic [172], lipid-lowering [173], anxiolytic and antipyretic [137], antiophidic [174]	No

Iridaceae				

*Calydorea *sp./Jabotitana	Decoction/Rhizome	Analgesic [23]	Not found	Not found

Labiatae				

*Agastache mexicana* Kunth/Toronjil	Not found	Sedative [102]	Antihypertensive [175], vasorelaxant [176], anti-inflammatory and antinociceptive [177], antinociceptive [178], anxiolytic [179]	No

*Lavandula latifolia*/Lavanda	Oil	Stimulant [68]	Anxiolytic [180], antifungal [181], antioxidant [182]	Yes

*Origanum vulgare*/oregano	Infusion/Leaf	Sedative [68]	Antimicrobial [183] proapoptotic effect and cytotoxic [184], antiurolithic [185]	Yes

Lamiaceae				

*Coleus barbatus *Benth./Falso-boldo	Tisane mate/Leaf	Headache, calmative [56]	Hepatoprotective [186]	Yes

*Cunila microcephala *Benth.*/*Hortelã-miúdo, hortelã-pimenta, poejo	Decoction/Complete plant	Analgesic [23, 58]	Anti-inflammatory and antioxidant [187]	No

*Hyptis suaveolen*s Poit.*/*Samba-coité	Tea/Leaf	Headache [188]	Hypoglycemic and antioxidant [189], hepatoprotective and antioxidant [190], gastroprotective activity [191], neuroprotective and antioxidant [192], antifungal [193]	Yes

*Lavandula officinalis *Chaix & Kitt*/*Alfazema	Tea/Leaf, stalk	Calmative [49]	Antimicrobial [194], antioxidant [195], sedative and hypnotic [196]	No

*Leonotis nepetifolia *(L.) R. Br.*/*Cordão de São Francisco	Infusion, decoction/Leaf, branches	Sedative, headache [132, 138]	Antimicrobial [197], anti-inflammatory [198]	Yes

*Melissa *officinalisL./Erva-cidreira, melissa	Decoction/Leaf	Calmative, migraine, sedative [23, 36, 55, 58, 59, 87, 89, 90, 101, 102, 138, 199]	Anti-inflammatory [200], calmative [201], antioxidant [202], antigenotoxic and antimutagenic [203], neuroprotective [199, 204]	No

*Mentha arvensis *L./Hortelã-mentol	Tea/Leaf	Headache [188]	Antibacterial [205], antifungal [206], anti-inflammatory and sedative [207], peptic antiulcer [208]	Yes

*Mentha cf. suaveolens Ehrh.*/Hortelã, hortelã-grande	Decoction, maceration/Leaf	Calmative, Analgesic [23, 199]	Antifungal [209, 210], antioxidant [211], antibacterial [212]	Yes

*Mentha piperita *L./Hortelã, hortelã-roxo	Decoction/Complete plant	Analgesic [23, 102]	Antifungal [213, 214], antioxidant [211], anthelmintic [215], hypoglycemic and hypolipidemic [216], anticancer [217] analgesic [218]	No

*Mentha pulegium *L./Poejo	Decoction/Stalk	Calmative, sedative [47, 56, 58, 78]	Antioxidant [211], antimicrobial [219]	Yes

*Mentha *sp./Hortelã	Decoction/Stalk	Headache, Calmative [36, 47, 49, 55–57]	Anthelmintic [215]	Yes

*Mentha spicata *L./*∗*	*∗∗∗*	Headache [87]	Hypoglycemic and hypolipidemic [216], antioxidant [220], antiemetic [221]	Yes

*Mentha *×* villosa *Huds./Hortelã	Tea/Leaf	Headache [188]	Antifungal and antibacterial [222], antimicrobial and antioxidant [223], analgesic and antispasmodic [153]	No

*Ocimum basilicum *L./Alfavaca	Decoction, maceration/Leaf	Calmative, analgesic [23, 39]	Antidepressant and anticonvulsant [224]	Yes

*Ocimum gratissimum *L./l Louro	Tea/Leaf	Headache, calmative [49, 87, 188]	Anticonvulsant [225, 226], antifungal [227]	Yes

*Ocimum minimum *L./Manjericão	Maceration/Leaf	Headache [94]	Antiulcerogenic and antioxidant [35]	Yes

*Ocimum selloi *Benth./Alfavaca	Infusion, tea/Leaf	Calmative [138]	Antibacterial [219], analgesic and antidiarrheal [220]	Yes

*Origanum majorana*L./Manjerona	Decoction/Stalk	Calmative [56]	Antibacterial [228], antioxidant [49], antimetastatic and antitumor [229], antihyperglycemic and antihyperlipidemic [230]	Yes

*Plectranthus barbatus *Andr.*/*Boldo	Decoction, maceration/Leaf	Analgesic [23, 57, 60]	Cytotoxic [231], acetylcholinesterase inhibitor [232], antimicrobial [233]	Yes

*Plectranthus neochilus *Schlechter/Boldo do Chile	Infusion/Leaf	Headache [89]	Analgesic [234]	No

*Rosmarinus officinalis *L.*/*Alecrim	Decoction/Leaf	Analgesic, calmative [23, 39, 48, 58, 59, 102, 138, 235]	Antibacterial [236], antioxidant [237], antifungal [238], anticancer [239], antidepressant [240], analgesic [241], antioxidant, anti-inflammatory, metal chelation [242], prevention and treatment of dementia [243], neuroprotective [244]	No

*Salvia lachnostachys *Benth.*/*Melissa	Decoction/Leaf	Somniferous [23, 78]	Anti-inflammatory and analgesic [244]	Yes

*Salvia lavandulifolia *Vahl./Mariselva	Oil/*∗∗*	Nervous disorders [245]	Hypoglycemic [245], neuroprotective [246]	No

*Salvia officinalis *L.*/*Salvia, barcelona	Decoction/Leaf	Calmative, Analgesic [23]	Antibacterial [228], anti-inflammatory [247], antidiarrheal and antispasmodic [185], analgesic and anti-inflammatory [248]	No

Lauraceae				

*Cinnamomum zeylanicum *Breyn./Canela	Infusion, maceration/Stalk	Calmative [39]	Antifungal [249] antimicrobial [250], antioxidant [251], antidiabetic [252]	Yes

*Nectandra megapotamica * (Spreng.) Mez/Canela-preta	Infusion/leaf	Calmative [253]	Anesthetic [254]	Yes

Leguminosae				

*Acosmium subelegans (Mohlenbr) Yakovl*/Perobinha do campo	*∗∗∗*	Sedative, epilepsy and nervous exhaustion [255]	Depressant effect SNC and anticonvulsant [255]	No

*Hymenaea courbaril* L./Jatobá	Infusion, maceration/Bark, fruit	Sedative [132]	Not found.	Not found

*Tamarindus indica*/Tamarindo	Compress, bathe, infusion/Stalk, leaves, fruit	Treatment of fever, stomach upset, diarrhea, jaundice and as skin cleansers [256], inflammation, urinary tract infection and laxative [257], headache and stress [258]	Antibacterial [256], antihelminthic [257], antioxidant [259], antinociceptive [260], analgesic and anti-inflammatory [261], antihistaminic and antianaphylactic [262], antiulcer [263]	No

Liliaceae				

*Allium sativum *L./Alho	*∗∗∗*	Headache [59]	Hypotensive [264], synergism with antibiotics [265], antioxidant [266]	Yes

Malpighiaceae				

*Banisteriopsis* *caapi*/Mariri, ayahuasca	Decoction, infusion/vine	Hallucinogen, emotional and cognitive sensory changes, psychoactive [267–269] aid in treatment of abuse of other Psychoactives [270]	Hallucinogen [271] inhibiting the reuptake of serotonin, in addition to inhibiting MAO [272]	No

*Galphimia glauca*/Amarilla	Maceration/Part air plants	Calmative [273]	Anxiolytic [273]	No

Meliaceae				

*Cedrela fissilis/*Cedro-rosa	Infusion/Bark	Headache [121]	Not found	Not found

Moraceae				

*Cannabis sativa*/maconha, marijuana, cânhamo	Oil, inhalation/Leaves, stalk, flowers	Treatment of pain, nausea and vomiting, multiple sclerosis and other neurological disorders, loss of appetite and eating disorders, Insomnia, anxiety and depression, neuroprotective action [274], antiemetic, appetite stimulant [275], clinical and experimental studies in the treatment of dementias [276], schizophrenia, antipsychotic, anxiety [277], antipsychotic [278]	Treatment of pain, nausea and vomiting, multiple sclerosis and other neurological disorders, loss of appetite and eating disorders, Insomnia, anxiety and depression, neuroprotective action [273], antiemetic, appetite stimulant [274], clinical and experimental studies in the treatment of dementias [275], schizophrenia, antipsychotic, anxiety [276], antipsychotic [277], psychoactive [278]	No

*Dorstenia brasiliensis *Lam.*/*Carapiá	Cataplasm/Rhizome	Anesthetic [67]	Anti-inflammatory [278]	Yes

Myrtaceae				

*Eucalyptus globulus *Labill./Eucalipto	Infusion, Bathe/Leaf	Headache [48]	Toxic effect [279], antibacterial [280, 281]	Yes

*Eugenia uniflora *L.*/*Pitangueira	Decoction/Leaf	Calmative [23, 282]	Antimicrobial and antioxidant [283], anti-*Trypanosoma cruzi *[206]	Yes

Orchidaceae				

*Vanilla planifolia *Jack. ex Andrews*/*Baunilha	*∗∗∗*	Calmative [67]	Not found	Not found

Oxalidaceae				

*Averrhoa Carambola *L./Carambola	Infusion/Leaf	Analgesic [99]	Analgesic [284]	No

Papaveraceae				

*Papaver somniferum*/Planta do ópio	*∗∗∗*	Analgesic and sedative [68]	Not found	Not found

Passifloraceae				

*Passiflora alata* Curtis/Maracujá	Fruit	Calmative [55, 59, 77, 90]	Sedative [285]	No

*Passiflora caerulea *L./Maracujá	Infusion/Part air plant	Sedative and calmative [91, 286]	Anxiolytic [287]	No

*Passiflora edulis *Sims./Maracujá	Tea/Leaf	Calmative and insomnia [39, 48, 74, 77, 78, 90, 99, 257]	Anxiolytic [288]	No

*Passiflora miersii *Mart./Maracujazinho	Infusion/Leaf	Calmative and antidepressant [67]	Not found	Not found

Pedaliaceae				

*Sesamum orientale *L./Gergelim	Seed/Juice	Anticonvulsant [99]	Hypoglycemic [289]	Yes

Phytolaccaceae				

*Petiveria alliacea *L.*/*Guiné, tira capeta	Decoction/Complete plant	Analgesic [23, 74, 99, 290]	Antimicrobial [291], antinociceptive, sedative, anticonvulsant and depressant [292]	Yes

Piperaceae				

*Pothomorphe umbellata *Miq.*/*Pariparoba	Infusion/Leaf	Headache [121]	Antioxidant [293], antitumor [294], antihelminthic [295]	Yes

Poaceae				

*Cymbopogon citratus*Stapf./Capim santo, capim limão	Decoction/Leaf	Calmative, analgesic and sedative [7, 23, 27, 36, 39, 47–49, 55–58, 74, 77, 78, 88–90, 99, 101, 102, 138, 296]	Anxiolytic, sedative and anticonvulsant [297]	No

Polygalaceae				

*Polygala paniculata *L.*/*Arnica	Decoction/Complete plant	Analgesic [23]	Analgesic and antidermatogenic [298], antinociceptive and gastric cytoprotective activity [299]	No

Polygonaceae				

*Homalocladium platycladum *Bailey*/*Carquejinha	Decoction/Stalk	Analgesic [23]	Antibacterial [300], analgesic, anti-inflammatory [301]	No

Rosaceae				

*Rosa centifolia *L.*/*Rosa branca	Decoction/Leaf, flower	Analgesic [23]	Anti-inflammatory and antiarthritic [302], antioxidant [303], antiulcer and cytoprotective [304]	No

*Sanguisorba minor *Scop./Pimpinela	Tea/Leaf, flower	Calmative [102]	Inhibitory action of acetylcholinesterase [305]	Yes

Rubiaceae				

*Coffea arabica *L./Café	Cataplasm/Leaf	Headache [101]	Antioxidant [306], antioxidant and stimulant [307]	Yes

*Cinchona officinalis *L.*/∗*	Decoction/Bark	Analgesic [23]	Not found	Not found

*Psychotria viridis*/chacrona, ayahuasca	Infusion/Leaves	Hallucinogen, emotional and cognitive sensory changes, psychoactive [267–269] aid in treatment of abuse of other Psychoactives [268]	Hallucinogen [308]	

*Alibertia *sp./Marmelo	Decoction, infusion/Root, fruit	Calmative [118]	Not found	Not found

Rutaceae (5)				

*Casimiroa edulis *Llave & Lex./Zapote blanco	*∗∗∗*	Sedative [102]	Vasodilator [309, 310], anticoagulants and antimicrobial [310], anxiolytic [311], anxiolytic and antidepressant [312]	No

*Citrus aurantium *L./Laranja	Decoction/Bark	Headache and calmative [36, 48, 56, 59, 78, 90, 194]	Low toxicity [313], anxiolytic [314, 315]	Yes

*Citrus limon *(L.) Burm. f./Limão-galego,	*∗∗∗*	Calmative and sedative [90, 194]	Neuroprotective activity and anticonvulsant [316]	Yes

*Citrus sinensis (*L.) Osbeck	Infusion/Leaf	Calmative, analgesic and sedative [23, 27, 49, 74, 99, 138]	Antioxidant, antithyroid and antihyperglycemic [317]	Yes

*Ruta graveolens *L.*/*Arruda	Decoction, maceration/Leaf	Calmative and headache [23, 39, 48, 57, 60, 102]	Antimicrobial [318], antioxidant [319], antitumor [320], antinociceptive, anti-inflammatory and antipyretic [321]	Yes

Solanaceae				

*Atropa belladonna *L.*/*Beladona	Decoction/Leaf	Calmative [23]	Healing [322]	Yes

*Cestrum sendtnerianum *Mart./Guiné-do-campo	Infusion, Decoction/Leaf	Sedative [67]	Not found	Not found

*Solanum americanum *Mill./Maria-pretinha	Decoction/Leaf, Stalk	Sedative, Analgesic [45, 67]	Antifungal [323], antioxidant and anticancer [324]	Yes

*Solanum cernuum *Vell/Pata de mono	*∗∗∗*	Calmative [87]	Antiulcerogenic [325]	Yes

Umbelliferae				

*Anethum graveolens*/Eneldo	*∗∗∗*	Sedative [68]	Antifungal [326], anticonvulsant [327], anti *Helicabator pylori *[328], decreased fertility rate [329], participates in the regulation of Diabetes Mellitus [330]	Yes

*Coriandrum sativum*/Cilantro	Infusion/Leaf, fruit	Stimulant [68]	Antioxidant [40], sedative and muscle relaxant [43], antibacterial [331], antiarthritic [332], anti-inflamatory [41], antifungal [333], hypoglycemic and hypolipidemic [334]	Yes

*Petroselinum hortense/*Salsa da horta	*∗∗∗*	Sedative [107]	Diuretic and hypotensive [335]	Yes

Urticaceae				

*Urera baccifera *(L.)/Urtiga	Leaf	Analgesic [36]	Antioxidant [336], anti-inflamatory [337]	No

Verbenaceae (6)				

*Aloysia citrodora *Palau/Erva luíza	*∗∗∗*	Calmative [74, 286]	Not found.	Not found

*Aloysia triphylla *Royle/Cidrão	*∗∗*/Leaf	Sedative [55, 235]	Treatment of intestinal disorders [338], anti *Trypanosoma Cruzi *[339], anti-*Helicobacter pylori *[328], antibacterial [340], spasmolytic and anti-inflammatory [341], antinociceptive [244]	Yes

*Lantana camara *L./Camará	Infusion, Decoction/Leaf	Headache [132]	Antibacterial [342], antioxidant [343], anxiolytic [344]	Yes

*Lippia alba *(Mill.) N.E. Br./Erva-cidreira	Leaf/Infusion	Headache and calmative [39, 49, 55, 58–61, 75, 77, 94, 97]	Antimicrobial [345], antispasmodic [346], anxiolytic [347], anesthetic [348]	No

*Lippia gracillis Schauer*/Alecrim da serra	Infusion/Leaf	Headache [132]	Antimicrobial [349], antitumor [350], anti-inflammatory and healing [351]	Yes

*Verbena cf. minutifolia Phil./∗*	Decoction/Complete plant	Analgesic [23]	Not found	Not found

Violaceae				

*Viola odorata *L./	*∗∗∗*	Sedative [87]	Antitumoral [352], antioxidant and antibacterial [353], antimicrobial [354], vasodilator and antidyslipidemic [355]	Yes

Zingiberaceae				

*Alpinia zerumbet *(Pers.) Burtt & Smith/Colônia	Decoction/Leaf	Calmative [39, 48, 49, 101]	Hypotensive [356], vasodilator [357], antioxidant [358]	Yes

*Zingiber officinale *Rosc./Gengibre	Decoction/root	Analgesic and headache [23, 57, 78]	Antioxidant [359], antihyperglycemic [360], antibacterial [361], androgenic [362]	Yes

*Costus brasiliensis *Schum./Cana-de-macaco	Not found	Calmative [67]	Not found	Not found

^*∗*^It is the popular name that was quoted. ^*∗∗*^It was not mentioned how to prepare. ^*∗∗∗*^It is the portion used or how to prepare that was quoted.
